# Participation in physical activity by adolescents with physical disability: Protocol for a national participation snapshot and Delphi consensus study (“Youth Experience Matters”)

**DOI:** 10.12688/hrbopenres.13741.2

**Published:** 2024-04-04

**Authors:** Karen Brady, Damien Kiernan, Elaine McConkey, Eva O'Gorman, Claire Kerr, Suzanne McDonough, Jennifer Ryan, Ailish Malone

**Affiliations:** 1 School of Physiotherapy, Royal College of Surgeons in Ireland University of Medicine and Health Sciences, Dublin, Ireland; 2 Central Remedial Clinic, Dublin, Ireland; 3 School of Nursing and Midwifery, Queen's University Belfast, Belfast, Northern Ireland, UK

**Keywords:** disability; participation; physical activity; Delphi; physical disability; adolescents

## Abstract

Young people with physical disability experience challenges to being physically active. To attain the health benefits of physical activity (PA) and sustain engagement, it is essential that participation is meaningful and enjoyable. This study aims to describe current participation in PA by adolescents with physical disability in Ireland, and to establish consensus on their priorities for enhancing physical activity participation. A parallel convergent mixed methods study will be undertaken, comprising a national cross-sectional quantitative assessment of PA participation (“Participation Snapshot”) and Delphi consensus study (“Delphi”). Adolescents (n=100) aged 13–17 years with a physical disability will be invited to take part. The Participation Snapshot primary outcome is the Children’s Assessment of Participation and Enjoyment (CAPE). Contextual factors including underlying medical diagnosis, demographics, mobility (Functional Mobility Scale), hand function (Manual Ability Classification System) and health related quality of life (Child Health Utility 9D) will also be collected. The Delphi will comprise two to four survey rounds, until consensus is reached. Round 1 consists of a bespoke survey, designed and piloted with a public and patient involvement (PPI) panel, with open-ended questions and Likert scales inviting contributions from adolescents on their prior experience and ideas to enhance participation. Responses will be analysed using inductive thematic analysis to construct items and themes, which will then be deductively mapped to the “F-words” and the family of Participation-Related Constructs frameworks. These items will be presented back to participants in subsequent rounds for selection and ranking, until consensus is achieved on the “top 10 priorities” for enhancing PA participation. The project team and PPI panel will then co-design dissemination material and identify targets for dissemination to relevant stakeholder or policy groups. The findings will provide a basis for developing interventions aiming to enhance future PA participation for adolescents with physical disability.

## Introduction

The benefits of regular physical activity (PA) are undisputed. Regular PA can positively influence cardiorespiratory fitness, muscular fitness, cardiometabolic health, bone health and cognitive function for children and adolescents ( World Health Organisation, 2020). Children and young people with disability stand to benefit significantly from sustained PA participation, as demonstrated by the following evidence. Engagement in PA, as measured by the self-reported Physical Activity Questionnaire for Adolescents (PAQ-A), was found to predict quality of life and happiness in adolescents with Cerebral Palsy (CP) ( Maher *et al.*, 2016). Evidence from high-quality randomised controlled trials (RCTs) has shown that young people with disability can gain cardiovascular fitness and strength from participation in PA ( Taylor *et al.*, 2013), particularly PA at a moderate-to-vigorous intensity ( Verschuren *et al.*, 2014). This demonstrates high potential to benefit from PA across multiple domains of health ( Smith *et al.*, 2022).

A recent proposed update to Ireland’s National Physical Activity Guidelines recommend all children and young people with disability to engage in moderate to vigorous activity at least 60 minutes every day, and to include muscle strengthening, flexibility and bone-strengthening exercises three times a week ( Murtagh *et al.*, 2024), though it is noted that health benefits can still accrue with lower amounts. This is reflected in the UK Chief Medical Officer’s guidelines, specific to children and young people with physical disability, which recommend 120 to 180 minutes of aerobic physical activity at a moderate-to-vigorous intensity each week, with strength and balance activities on at least three days per week ( Smith *et al.*, 2022). Despite the guidance and potential benefit, young people with physical disability do not achieve the recommended PA targets and are much less active than their non-disabled peers ( Carlon *et al.*, 2013; Ryan *et al.*, 2015; Verschuren *et al.*, 2016). A systematic review, comprising six studies with data from 343 young people with CP and 706 typically-developed peers, found that young people with CP participated in 13–53% less PA and were sedentary for twice the recommended amount ( Carlon *et al.*, 2013). It appears that the COVID-19 pandemic has also impacted PA participation for people with disability: an online public engagement, non-peer reviewed survey of 1,842 disabled people found that just 30% of respondents felt encouraged to return to PA after the pandemic ( Activity Alliance, 2022). The effects of physical inactivity in this population are profound. A recent meta-analysis found higher incidence and prevalence of metabolic, cardiovascular diseases and non-communicable disease, for which physical inactivity is a risk factor, in people with CP ( Ryan *et al.*, 2023). There is an urgent need to address physical inactivity across all populations ( Santos *et al.*, 2023) but particularly in young people with physical disability, who already have lower baseline levels of cardiorespiratory endurance and muscle strength and may be at risk of declining mobility and musculoskeletal complications ( O'Sullivan *et al.*, 2020).

Physical activity can be achieved through incidental physical activity or through planned participation in organised, structured exercise often with other people. Participation, defined in the International Classification of Functioning, disability and health (ICF) ( World Health Organisation, 2001) as “involvement in a life situation”, is aptly construed as “both a means and an end” ( Imms *et al.*, 2017). The “F-words” conceptualisation of the ICF captures participation under the F-word “friends”, to denote the social interactions from participating with others ( Rosenbaum & Gorter, 2012). Participation could be crucial to sustaining PA. Adolescence is a significant time of change in participation, when adolescents with physical disability shift away from recreational and physical activities towards more social participation ( Imms & Adair, 2017). In a recent systematic review, McKenzie *et al.* (2021) reported the social and physical environment as strong influences on PA participation in young people and adults with childhood-onset disabilities. They found that social connectedness, social support and the physical environment were fundamental to ‘finding the right balance’, which could tip the balance in favour of being physically active or not. This is an important consideration in informing research into interventions to improve physical activity, as many exercise programmes are not sustained in the medium- to long-term. Understanding how young people participate in PA is crucial to any intervention that aims to enhance and enable sustained PA participation. There is no one-size-fits-all approach to participation ( Shields & Synnot, 2016).

To determine how a participation-based approach could improve physical activity, it is necessary to measure current participation and understand the perspectives of adolescents themselves on the factors that could enhance participation.

## Aim

The aims of this study are:

1. To describe current participation in physical activity by adolescents with physical disability in Ireland;
2. To establish consensus among adolescents with physical disability regarding their priorities for enhancing their physical activity participation.

Specific objectives are:

1. To report participation in physical activity over the previous four months by adolescents with physical disability
2. To compare participation between categories of gender, mobility, geography and diagnosis;
3. To classify factors that enable or constrain participation in physical activity from free text responses, using the family of participation-related constructs (fPRC) framework
4. To describe adolescents’ perceived importance of each factor using data from Likert scales
5. To achieve consensus on top 10 priorities for enhancing physical activity participation.

## Methods and analyses

**Figure 1.**
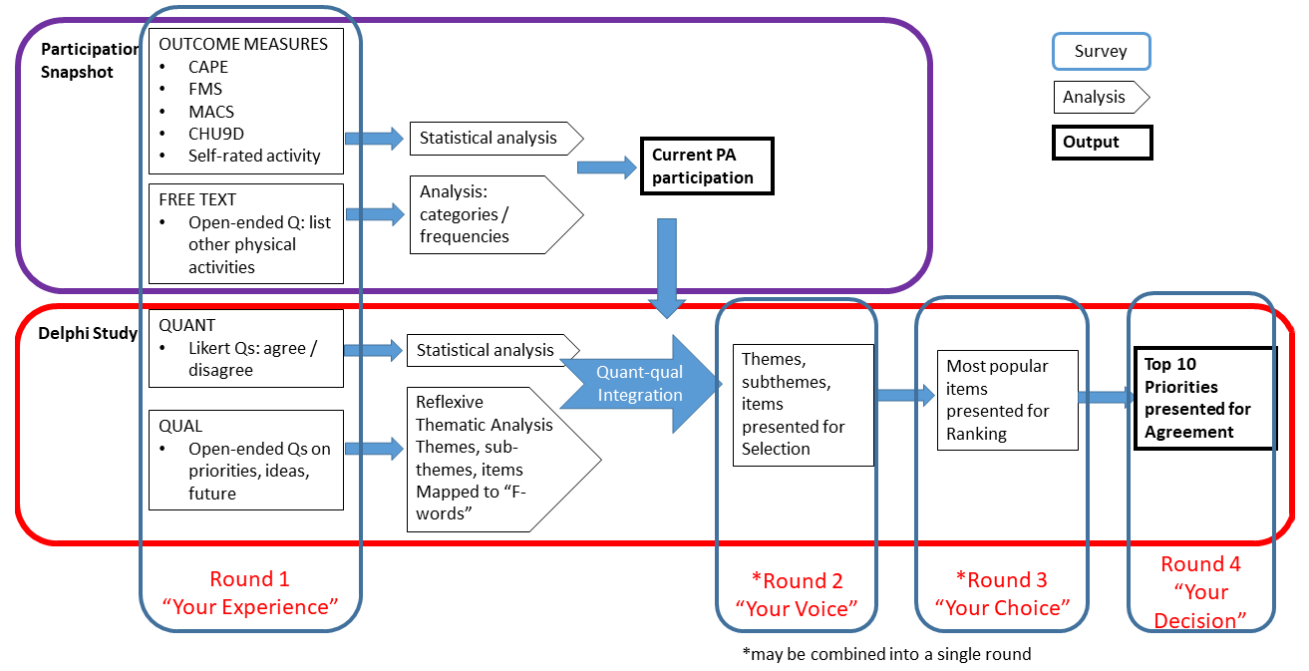
Participation Snapshot and Delphi Study: Data collection, analysis and integration.

A cross-sectional convergent mixed methods study will be conducted. Figure 1 outlines the phases.

### Participation Snapshot Study

The Participation Snapshot is a cross-sectional study, comprising an online, paper and/or interview led survey, aiming to describe and quantify current participation in PA by adolescents with physical disability in the Republic of Ireland. This will be achieved through quantitative data from standardised scales and Likert scales, supplemented by open-ended questions exploring current participation. The Children’s Assessment of Participation and Enjoyment (CAPE) is the primary outcome measure ( King *et al.*, 2007).

### Delphi Study

The Delphi study is a mixed methods study aiming to determine the Top 10 Priorities from the perspectives of adolescents themselves on the factors that could enhance their participation in PA. The Delphi technique is a well-established approach to achieving consensus between experts in a field, through an iterative process using questionnaires ( Barrett & Heale, 2020; Stennett *et al.*, 2018). In the Delphi, young people with physical disability will be considered the experts in their lived experience. A minimum of two rounds and maximum of four rounds will be undertaken, until a clear consensus is achieved.

## Public and Patient Involvement (PPI)

A PPI panel, comprising three young people who have a physical disability and three parents/guardians of young people who have a physical disability, works alongside the project team at all stages from inception to dissemination. They helped identify the research question, inputted in the development of the proposal and the methodological approach. They helped develop Round 1 surveys, particularly with regard to language, order and visual appearance, and piloted initial drafts of the online and paper versions. They guided the project team on recruitment strategies and contributed video and photo footage to the promotional material on the project website.

The PPI panel will similarly input into the design of subsequent Delphi rounds. When consensus is reached on the “Top 10 Priorities”, a focus group will be convened, at which the project team and PPI panel will co-design dissemination materials (including infographics, videos and other media) and identify target audiences with whom to share these outputs.

## Ethics

The study has been approved by the host institution RCSI University of Medicine and Health Sciences Research Ethics Committee (REC202207012) and two lead national disability agencies, the Central Remedial Clinic (REC12118) and Enable Ireland (RA88). As all participants will be under the age of 18 at study commencement, parent/guardian consent will be required, in addition to young person assent. The participant/parent dyad will receive a Parent Information Leaflet, Young Person’s Information Leaflet, and consent form. Study information and materials will also be available on the study website. Participants who turn 18 during the timeframe of the Delphi study will be re-consented using a Participant Consent Form seeking informed consent, to be completed digitally (for online participants) or written on paper (for paper-based completion).

## Participants and eligibility

Participants will be young people aged 13-17 years at study commencement with a primary diagnosis of physical disability. The definition, from a diagnostic perspective, of primary diagnosis of physical disability has been thoroughly considered in preparing this protocol. The following inclusion criteria will be applied in line with similar diagnostic categories reported by Anaby *et al.* (2020).

1. A primary diagnosis of one of the following causes of physical disability
  ▪ Cerebral palsy (CP)
  ▪ Spina bifida (SB)
  ▪ Spinal cord injury (SCI) of American Spinal Injuries Association Impairment Scale (AIS) A-D, any neurological level
  ▪ Acquired brain injury caused by stroke or non-invasive cerebral tumour or non-progressive, treated vascular abnormality
  ▪ Traumatic brain injury causing persistent physical disability
  ▪ Skeletal abnormality diagnosed pre- or perinatally, namely achondroplasia or osteogenesis imperfecta
  ▪ Brachial plexus injury (BPI) or obstetric brachial plexus palsy
  ▪ Limb loss
  ▪ Neuromuscular disorder
  ▪ Other disorders as reported by parent/child dyads in the questionnaire
2. Able to understand and contribute to questionnaires, either independently or with the support of a family member or therapist.

**For feasibility reasons, the following exclusion criteria will apply:**

1. Intellectual disability of such severity that, even with supports, the parent judges that the adolescent is unable to understand or contribute meaningfully to the questionnaire.
2. Inability to read or write in English, even with support.

## Sample size

The target sample size is 100 participants. There is no current national register that captures the total population with the inclusion criteria as outlined, so the sample size was estimated from data from the National Physical and Sensory Disability Database (NPSDD) report 2018 ( NPSDD, 2017) and the Irish national Census in April 2016 (the latest available data at the time of planning the study in September 2021). Extrapolating from Census 2016, 411,519 people living in Ireland would be age 13–17 in 2022 ( CSO, 2016). Considering the known prevalence of some of the included conditions, including CP (1.6 per 1,000 live births) ( McIntyre *et al.*, 2022), SB (1 in 10,000 births in Ireland 2009–2011) ( McDonnell *et al.*, 2015) and BPI (1.7 per 1,000 live births) ( Walsh *et al.*, 2011), we estimate an overall prevalence of physical disability with eligibility for this study of 0.5%, or 2,057 adolescents. This estimate aligns with the NPSDD, of which 10% of its 20,676 registered service users were aged 13–17 years. Taking 2,000 eligible participants as the target population, recruiting 92 participants will give a confidence interval of ±10% for calculation of the Participation Snapshot ( Conroy, 2021). There is no recommended sample size for a Delphi study; a Delphi study on exercise priorities for people with multiple sclerosis (MS) included a comparable sample size of 100 ( Stennett *et al.*, 2018). Therefore, we aim to recruit 100 participants to the Participation Snapshot and Delphi Studies, estimated to be 5% of the target population. 

## Recruitment

Participants will be recruited in two ways: firstly, through clinical services, and secondly through the wider community and public. Recruitment through clinical services will be led by 15–20 physiotherapists and occupational therapists who work within Children’s Disability Network Teams (CDNTs) and national specialist services in Ireland and have been identified as “gatekeepers” for their teams. We considered the geographical distribution in identifying the study gatekeepers. The lead clinical site, based in Dublin, serves young people from all over Ireland in its specialist services, and additionally has smaller clinics in the south east and west of Ireland. The other clinical partner provides children’s services in 11 of 26 counties, and in all four provinces. Gatekeepers will share information about the study with potentially eligible parent/young person dyads through the course of their usual care or therapy interactions. Additionally, the database at the lead clinical site, the Central Remedial Clinic, will be screened to identify eligible service users who attended in the 18 months prior to data collection. The nominated parents/guardians of these eligible service users will receive an invitation to participate by post. Reminders will be sent three–six weeks from initial contact.

**Figure 2.**
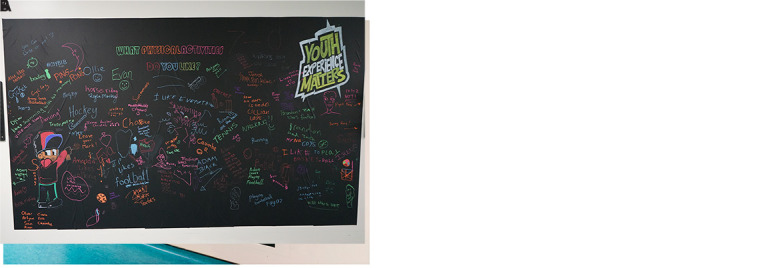
“Interactive wall” to raise awareness and promote Youth Experience Matters.

Local initiatives have been undertaken at the lead clinical site to promote the study. These include a screen to play the promotional video, placing pull-up banners at the clinic’s reception and waiting areas, and the “interactive wall” ( Figure 2) inviting contributions from young people on topics relevant to their participation in PA. Photos from the “interactive wall” will be shared on social media periodically to sustain interest. All promotional materials will be updated to denote the launch of the second and subsequent rounds.

Recruitment through the public and wider community will be undertaken by engaging with sporting organisations, disability charities and support groups, and the media. A promotional video and poster will be prepared for sharing *via* social media, with links to the study webpage and recruitment materials. Paper copies will be provided to supporting community organisations, charities and support groups, for onward sharing.

## Data collection

Data will be collected either online *via* the study website, *via* paper copies at participating clinical sites and community organisations, or *via* interview (in person or video / telephone call). The online survey will be hosted on the Research Electronic Data Capture Data Management platform (REDCap) platform, distributed *via* the project’s website. Paper-based surveys will be distributed at participating clinical sites and community organisations. Participants can complete these independently or with support from a parent, guardian or other nominated person, with the stipulation that the responses reflect the young person’s views and not those of the supporting person. Participants can request completion *via* interview, in person or phone, by contacting the project team at an email address provided in the Participant and Young Person Information Leaflets (see *Extended data*) 

## Outcome measures

In this convergent mixed methods design, quantitative and qualitative data will be captured simultaneously.

### Participant characteristics

The following participant demographics will be collected for contextual purposes: age, gender (self-reported), diagnosis, residential area (county), any assistive equipment used to participate in daily activities. Participants will also be asked about any other additional needs that may impact their participation including medical conditions, learning difficulties, neurodiversity, communication difficulties, and recent medical or surgical intervention. The Functional Mobility Scale (FMS) ( Graham *et al.*, 2004) and Manual Ability Classification System (MACS) ( Eliasson *et al.*, 2006) will be captured to describe mobility and hand function, respectively.

### Participation Snapshot

The Participation Snapshot will capture current participation in PA, using a quantitative standardised outcome measures (the Children’s Assessment of Participation and Enjoyment (CAPE) ( King *et al.*, 2007) and self-reported categorical frequency of PA participation) and supported by open-ended questions inviting free text qualitative responses. For feasibility, the Participation Snapshot will be distributed with Round 1 of the Delphi study, presented with subheadings to distinguish the sections. 

The measures used to quantify PA participation have been selected to align with the attendance, involvement, preferences, context and environment constructs of the family of participation- related constructs (fPRC) ( Adair *et al.*, 2018; Imms *et al.*, 2017). The primary outcome measure is the CAPE ( King *et al.*, 2007), a 55-item questionnaire designed to examine how children participate in a range of everyday activities outside of school. As this study focuses on physical activity participation only, we used a 16-item subsection of the CAPE with the following items pertaining to physical activity: items 16–21 from the section Organised Sports, items 31–41 excluding items 38 (gardening) and 39 (fishing) from the section Active Physical Recreation, and item 24 (learning to dance) from the section Other Skill-Based Activities. This was based on the methods of Woodmansee *et al.* (2016), who measured participation in physical activity in 163 children (6–17 years) with physical, intellectual, or sensory disability, using the same 16 CAPE items.

Health related quality of life HRQoL as a secondary outcome will be measured using the Child Health Utility 9D CHU-9D ( Ryan *et al.*, 2020; Stevens, 2009).

### Delphi study

The Delphi study will follow a similar format to that described by Stennett *et al.* (2018), with up to four Rounds to achieve consensus on young people’s priorities for enhancing their participation in PA. The surveys in each round will be designed with the PPI panel and piloted by three to five young people age 13-17 before data collection commences.

Round 1 (see *Extended data*) is predominantly qualitative, captured through open-ended questions inviting free text responses, with some quantitative data from Likert scales to gauge agreement or disagreement with statements pertaining to PA participation. Participants have the option to contribute a PhotoVoice ( Wang & Burris, 1997) by including an image representing their participation in PA (the image must not contain identifiable information or individuals). The quantitative and qualitative data will be integrated at the end of Round 1, to create the items presented for consensus in Round 2.

Round 2 will take the form of item selection. We will ask participants to indicate the importance of each item with sliding or Likert scales. Round 2 will be analysed using descriptive statistics, specifically, frequency with which an item was positively rated on the sliding or Likert scale, and mean Likert score. We set the threshold for consensus at 70% agreement, meaning that at least 70% of participants must give an item a positive score for it to be considered a priority. Depending on the number of items exceeding this threshold, and the aggregate Likert scores for these items, it may be feasible to rank selected items in Round 2, thereby combining Rounds 2 and 3 into a single Round 2. This decision will be taken by the PPI panel and Project Management Team. A final round will present the top 10 priorities for agreement. Consensus will be defined as at least 75% agreement with the top 10 priorities in the final round.

**Figure 3.**
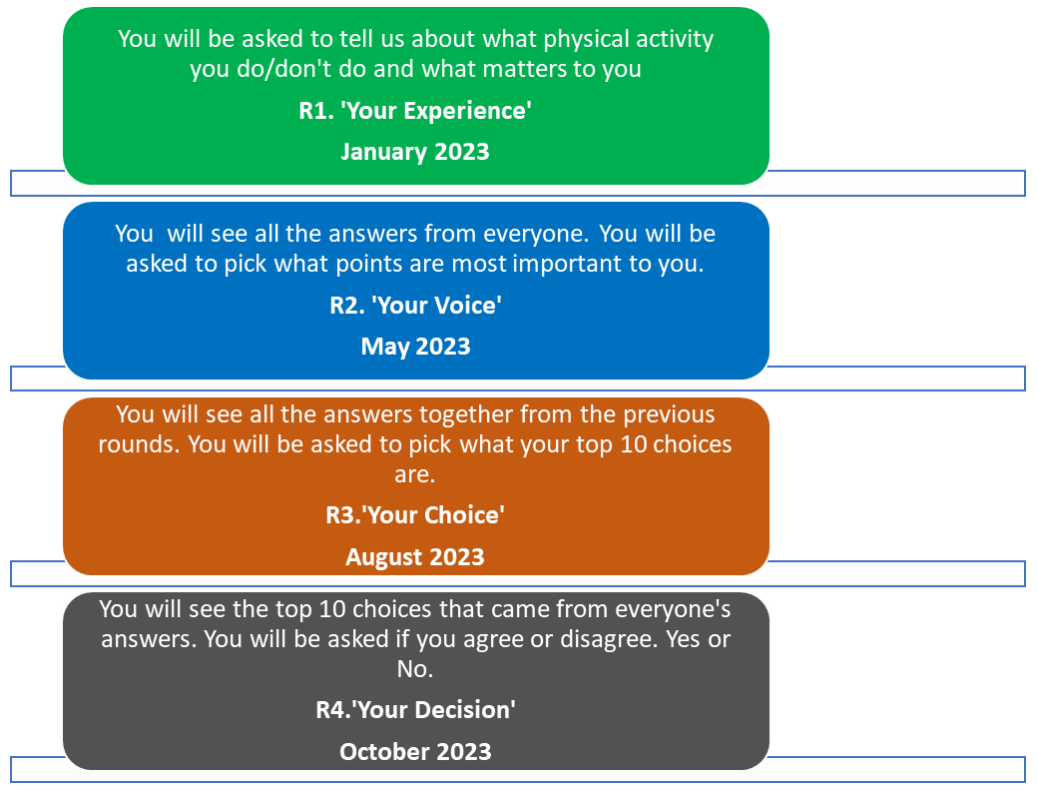
Delphi rounds descriptions and timelines.

The phases and timeline of the project are outlined in Figure 3. A detailed version of each of the proposed Delphi rounds is outlined in Figure 1. The study will take place between March 2023 and March 2024. Recruitment is ongoing since March 2023.

## Participant retention

The following strategies will be administered to help promote completion of the Participation Snapshot and Delphi Study, participant retention in the Delphi Study, and reduce missing data and attrition rates. Online questionnaires will utilise REDCap survey platform’s inbuilt functionality to require completion of questions and a progress bar with positive feedback encouraging completion, for example, “You are over half way there! Just two questions to go”. For retention to the second and subsequent survey rounds, participants will be contacted *via* email/post with reminders to complete the next round of the questionnaire. Awareness-raising initiatives on social media and at clinical sites (described in ‘Recruitment’ section) aim to promote sustained interest.

## Participant withdrawal

Participants may withdraw from the study at any time without consequences. Participants and parents will be informed of their right to withdraw at any time in the study information leaflets. Any de-identified data collected up to the time of withdrawal will be retained and included in analysis. The reason for withdrawal from the study will be documented, if known.

## Statistical analysis

The Participation Snapshot will be analysed by descriptive statistics and graphical analysis, in accordance with the objective to describe current participation in PA by young people aged 13-17 across Ireland. Data will be presented disaggregated by gender. As a secondary analysis, we will describe the dependent variables CAPE (primary) and CHU-9D (secondary) by relevant categories of participant characteristics (age, gender, condition, geographical location, mobility *via* FMS, hand function *via* MACS).

Delphi Round 1 will be analysed using reflexive thematic analysis, an interpretative approach that facilitates the identification and analysis of patterns or themes in a given data set ( Braun & Clarke, 2021a; Braun & Clarke, 2021b). The epistemological consideration will be constructionist. As such, the team will consider not just the recurrence, but also the meaning and meaningfulness of the issues arising within the dataset, in determining the themes. An experiential orientation to data interpretation will be adopted to prioritise the respondents’ own accounts of their experience, rather than imposing any expected findings ( Braun & Clarke, 2021a). Analysis will be predominantly inductive, with open coding to represent the meaning as communicated by respondents. Reflexivity notes will be kept at each stage. The software NVivo 12 (QSR International, Melbourne, Australia) will be used to organise the text in the data set.

A collaborative approach to analysis will be undertaken, with two members of the research team undertaking step 1 (familiarisation) and step 2 (coding) independently. They will then meet to generate initial themes (step 3) through a process of peer debriefing, leading to the creation of a thematic map of themes and subthemes. The “F-words” conceptualisation of the ICF ( Rosenbaum & Gorter, 2012) and the fPRC ( Adair *et al.*, 2018; Imms *et al.*, 2017). will be used as frameworks for the thematic map. At step 4, themes and the thematic map will be reviewed by the project management team, followed by the creation of a final map and Round 2 of the Delphi survey, which will be piloted by the PPI panellists.

As outlined under subheading “Delphi study”, Round 2, and subsequent rounds, will be analysed using descriptive statistics, specifically, frequency with which an item was positively rated on the sliding or Likert scale, and mean Likert score.

Demographic and contextual data will be described for respondents of each survey round. To ensure findings can be interpreted in context, participant characteristics (e.g., age, gender, diagnosis, FMS and MACS levels) will be compared descriptively between rounds to explore for differences in the profile of respondents across the Delphi rounds.

## Data management

A Data Management Plan has been developed. All data will be stored securely on a shared drive with restricted access, with multifactor authentication in place for additional protection. Data entered electronically via the REDCap platform will be downloaded to a secure study database. Paper surveys will be manually entered in the study database and hard copies will be stored in a secure filing cabinet. Data entry and validation will be a continuous process. Any identifying information (namely parental or participant email) will be stored separately from the point of user input (REDCap) or receipt of survey and consent (paper versions) to preserve participant anonymity. Newly generated study data will be irrevocably anonymised from the time of entry, as no “key” will be generated.

## Dissemination and knowledge translation

The results of this study will be shared within the scientific community through peer reviewed publications and national and international conferences. The project team and PPI panel will co-design dissemination materials (including infographics, videos and other media) and identify meaningful ways of sharing the results of this study, exploring possibilities such as a video voiced by young people with disability, infographics targeting the relevant stakeholders, PhotoVoice ( Wang & Burris, 1997), social media and opportunities to influence policy and practice. We anticipate that adolescents’ priorities for enhancing physical activity participation could have implications for intervention development, research, policy, education, and clinical practice.

## Data Availability

No underlying data are associated with this article. Figshare: Youth Experience Matters: Parent Information Leaflet https://doi.org/10.6084/m9.figshare.23278283.v1 This project contains the following extended data: AD6791_YEM_Parent_A5Booklet_V6.pdf Figshare: Youth Experience Matters: Young Person Information Leaflet https://doi.org/10.6084/m9.figshare.23278316.v1 This project contains the following extended data: AD6791_YEM_YoungPerson_A5Booklet_V3.pdf Figshare: Youth Experience Matters: Promotional material https://doi.org/10.6084/m9.figshare.23278370.v1 This project contains the following extended data: AD6791_YEM_Pull-Up-V1.pdf Figshare: Youth Experience Matters: Round 1 survey (Parts 1 and 2) - for information only (not for completion by participants.) https://doi.org/10.6084/m9.figshare.23280209.v1 This project contains the following extended data: YEM_SurveyR1_reproduced.pdf Figshare: Youth Experience Matters Data Management Plan 02 May 2023 https://doi.org/10.6084/m9.figshare.23277638.v1 This project contains the following extended data: HRCI-HRB-2022-006_Youth_Experience_Matters-DMP_v1.2_02May2023.pdf Data are available under the terms of the Creative Commons Attribution 4.0 International license (CC-BY 4.0). The Participation Snapshot will be reported in accordance with the STROBE guidelines ( von Elm *et al.*, 2007). The Delphi survey will be conducted and reported in accordance with Guidance on Conducting and REporting Delphi Studies (CREDES) ( Jünger *et al.*, 2017).
