# Supplementary material for: Participation in physical activity by adolescents with physical disability: cross-sectional snapshot and future priorities (“Youth Experience Matters” protocol)
Source: HRB Open Res. 2024 Aug 20;6:38. Originally published 2023 Jul 31. [Version 3] doi: 10.12688/hrbopenres.13741.3 (PMC11292183; doi:10.12688/hrbopenres.13741.3)
Supplement: Peer review history

# Reviewer report: Reviewer response for version 3

**DOI:** 10.21956/hrbopenres.15323.r42012

**Published:** 2024-08-28

**Authors:** Maria João Carvalheiro Campos

**Affiliations:** 1 University of Coimbra, Coimbra, Portugal

Dear authors,

 I appreciate the significant effort that you have invested in improving the overall quality of the manuscript and addressing the reviewers' questions and concerns.

Is the study design appropriate for the research question?

Partly

Is the rationale for, and objectives of, the study clearly described?

Partly

Are sufficient details of the methods provided to allow replication by others?

Partly

Are the datasets clearly presented in a useable and accessible format?

Not applicable

Reviewer Expertise:

Disability, Inclusion, Physical Education, Adapted Sport

I confirm that I have read this submission and believe that I have an appropriate level of expertise to confirm that it is of an acceptable scientific standard.

# Reviewer report: Reviewer response for version 2

**DOI:** 10.21956/hrbopenres.15224.r41552

**Published:** 2024-07-31

**Authors:** Maria João Carvalheiro Campos

**Affiliations:** 1 University of Coimbra, Coimbra, Portugal

Dear authors,

 I find the paper interesting and in my opinion the topic is relevant and is suitable for indexing in HRB Open Research. Moreover, I appreciate the significant effort that you have invested in improving the overall quality of the manuscript and addressing the reviewers' questions and concerns. Unfortunately, I still have major concerns with the manuscript in its current form. Overall, it is my belief that significant restructuring still is necessary for the paper to meet the standards required for indexing in HRB Open Research.

Main Points for Revision/Clarification:

  

1. Although the author had already reduced the title, the title should be reformulated.
2. The purpose is very noble but also probably too ambitious in my perspective, since “there is much going on” on this protocol, with such a diverse population. I’m afraid that the results and their implications will be very limited.
3. Can this research address all the specific objectives within this study protocol?
4. Physical disability involves a wide range of functional profiles: mild to severe, degenerative and non-degenerative disorders, possible cognitive deficit, among several others conditions… did you take this diversity into account for the delphi study? And how many adolescents will participate?
5. Did the *Children's Assessment of Participation and Enjoyment (CAPE)* survey have been validated for the Irish population?
6. Authors finish the paper anticipating “that adolescents’ priorities for enhancing physical activity participation could have implications for intervention development, research, policy, education, and clinical practice.” Given the diversity of the sample I’m not sure that those guidelines are valid for ALL the adolescents with physical disability.

Therefore, I strongly recommend that the article should be edited carefully before resubmission.

Is the study design appropriate for the research question?

Partly

Is the rationale for, and objectives of, the study clearly described?

Partly

Are sufficient details of the methods provided to allow replication by others?

Partly

Are the datasets clearly presented in a useable and accessible format?

Not applicable

Reviewer Expertise:

Disability, Inclusion

I confirm that I have read this submission and believe that I have an appropriate level of expertise to confirm that it is of an acceptable scientific standard, however I have significant reservations, as outlined above.

# Response

**Published:** 2024-08-14

**Authors:** Ailish Malone

**Affiliations:** Royal College of Surgeons in Ireland, Ireland

We thank the reviewers for their feedback and suggestions. We now respond to each point in turn, detailing the changes we made to this new version.

1. *Although the author had already reduced the title, the title should be reformulated.*
  1. Response: We further condensed the title to *Participation in physical activity by adolescents with physical disability:* *cross-sectional snapshot and future priorities (“Youth Experience Matters” protocol)*
2. *The purpose is very noble but also probably too ambitious in my perspective, since “there is much going on” on this protocol, with such a diverse population. I’m afraid that the results and their implications will be very limited.*
  1. Response: We agree this was an ambitious protocol and to achieve it, we put in place an experienced team from two universities (RCSI and QUB), a specialist clinical site (the Central Remedial Clinic), and a dedicated and engaged public and patient involvement panel of three young people with disability and their parents. The project had a dedicated resource of a clinical scientist working full-time for 16 months.
  2. Regarding the results and implications of the project, it may be useful to note that the project has concluded. (This protocol paper was submitted to HRB Open Research in May 2023 and the peer review process has taken time.) The Delphi study, covering aim 2 and objectives 3-5, has been published in Developmental Medicine and Child Neurology (Brady et al., 2024). The participation snapshot, covering aim 1 and objectives 1-2, is being prepared for publication. We delivered oral presentations on the findings of both the Delphi and participation snapshot at the European Academy of Childhood Disability annual conference in Bruges, Belgium, in May 2024.
  3. The point on the diverse population recurs in point 4 and we have addressed it there.
  4. Regarding the results and implications being limited, we address this by emphasising the health and social benefits of participation in physical activity, and its importance to stakeholders, particularly young people and families as conveyed by our PPI panel. To illustrate the importance, in March 2024 we held a World Café event to share the study’s findings and had attendance from 40 stakeholders, including young people with disability and their families, healthcare professionals, community volunteers, national governing bodies for sport, teachers, and researchers. The purpose of the World Café was to identify “next steps” following the top 10 priorities. So far, this has led to two funding applications and a further PhD.  
3. *Can this research address all the specific objectives within this study protocol?*
  1. We were confident with our team and collaborations that these could be achieved and as noted in point 2, the project has concluded with aims and objectives achieved. One paper has been published and a further is underway.
4. *Physical disability involves a wide range of functional profiles: mild to severe, degenerative and non-degenerative disorders, possible cognitive deficit, among several others conditions… did you take this diversity into account for the delphi study? And how many adolescents will participate?*
  1. We recognise diversity within physical disability and the challenges in representativeness of a study sample. We put in place the following measures. First, we worked closely with our clinical sites. The research scientist, who was responsible for data collection, attended several clinics to support recruitment at our partner clinical sites, including clinics for spina bifida, neuromuscular conditions, and CP. Second, to mitigate selection bias, we tailored our recruitment materials to specifically state that we sought all perspectives, including people who do not enjoy physical activity or are not very active. Third, we worked with the wider community through support groups, charities, and national governing bodies. We invited them to share recruitment materials through their websites, social media, and newsletters. We did an interview on a local radio show and podcast to reach young people who were not attending services. Fourth, to avoid geographic bias, the clinical scientist physically travelled to events around Ireland where young people with disability were in attendance. This was made possible through our collaborations with charities and community organisations. We enlisted clinical colleagues from all four provinces and major cities as gatekeepers. Fifth, cognisant of barriers to participation with respect to communication impairment or cognition, we offered a one-to-one interview in place of the written or online survey and the clinical scientist maintained a visible presence at clinics and events to support this. We added a condensed account of these points under Methods | Recruitment.
  2. To account for the diversity of the cohort, we recorded detailed demographic and contextual data using validated outcome measures including the Functional Mobility Scale for mobility, the Manual Ability Classification Scale for hand function, and recorded diagnosis and support needs .We compared findings from both work packages across categories of gender, ambulation status, diagnosis, and geography. This is described under statistical analysis
  3. The target sample size was 100 adolescents. The published paper on the Delphi study describes the characteristics of the 116 adolescents who took part. We achieved a representative sample in terms of diagnosis (half had CP, the other half, a variety of diagnoses including neuromuscular, spina bifida, acquired brain injury, skeletal conditions, spinal cord injury), ambulation (just over one-third were wheelchair users), and geographical distribution (just over one-third were based in Dublin).
5. *Did the Children's Assessment of Participation and Enjoyment (CAPE) survey have been validated for the Irish population*?
  1. No, the CAPE has not been validated specifically for the Irish population. It was developed in Canada and has been reported in comparable populations in Australia. There is no validated outcome measure of participation that has been validated specifically for the Irish population. We recognised that there may be contextual and cultural differences impacting the findings of the CAPE. To investigate this, we included some open-ended questions to gather context. The CAPE asks about 16 categories of physical activity, including team sports, individual activities. We asked participants to specify the activity or code they took part in, so for example, under team sports, they could state whether this was football / soccer, rugby, or Gaelic games. This point has been added under Methods | Outcome measures | Participation snapshot.
6. *Authors finish the paper anticipating “that adolescents’ priorities for enhancing physical activity participation could have implications for intervention development, research, policy, education, and clinical practice.” Given the diversity of the sample I’m not sure that those guidelines are valid for ALL the adolescents with physical disability.*
  1. We are not directly developing guidelines based on the findings. We suggest that the findings could be useful, potentially at multiple levels from individual to research and policy. We agree this is a diverse cohort and in previous points described how we aimed to account for this diversity. A limitation of Delphi method is that a threshold for consensus (typically 75%) needs to be set and within a consensus, there will be a diversity of perspectives. Now that our study has been published, we can report that we achieved consensus on the top 10 priorities of over 82-93%. Even with a strong consensus, we acknowledge that 7-18% of adolescents have a different perspective. We advise that individual variation must still be considered and that a different method (in-depth qualitative interviews) would be needed to explore the reasons behind the findings. However, we also argue that priorities of the majority can be impactful. We deemed adolescents to be the experts in their own lived experience in determining their own priorities, and the priorities are created entirely by them (iterated from their free text responses in round 1). We argue that these are important findings and have implications for future practice, research and policy, to which the perspectives of adolescents with lived experience should be central.

References

 Brady, K., Cleary, R., O'Gorman, E., McDonough, S., Kerr, C., Kiernan, D., McConkey, E., Ryan, J., & Malone, A. (2024). Identifying the top 10 priorities of adolescents with a physical disability regarding participation in physical activity: A Delphi study. Dev Med Child Neurol. https://doi.org/10.1111/dmcn.15986

# Reviewer report: Reviewer response for version 2

**DOI:** 10.21956/hrbopenres.15224.r39022

**Published:** 2024-05-16

**Authors:** Caitriona Cunningham

**Affiliations:** 1 School of Public Health, Physiotherapy and Sports Science, University College Dublin, Dublin, Ireland

Revisions are appropriate and happy to approve  paper.

Is the study design appropriate for the research question?

Yes

Is the rationale for, and objectives of, the study clearly described?

Partly

Are sufficient details of the methods provided to allow replication by others?

Yes

Are the datasets clearly presented in a useable and accessible format?

Not applicable

Reviewer Expertise:

musculoskeletal health, promotion of  physical activity and exercise for prevention and management of disease, disability inclusion

I confirm that I have read this submission and believe that I have an appropriate level of expertise to confirm that it is of an acceptable scientific standard.

# Reviewer report: Reviewer response for version 1

**DOI:** 10.21956/hrbopenres.15031.r37696

**Published:** 2024-02-26

**Authors:** Kwok Ng

**Affiliations:** 1 Faculty of Education, University of Turku, Turku, Finland; 2 Institute of Innovation and Sports Science, Lithuanian Sports University, Kaunas, Lithuania; 3 University of Limerick, Limerick, County Limerick, Ireland

Title is very long. Suggest to shorten. Remove the youth experience matters, or combine the next two parts together.

**General comments**

- This is an interesting study and highlight ways to encourage people with disabilities to be the voices in research. It is a much needed area in research and can provide useful insights to help provide tailored programs for people with disabilities. The methods seem suitable for the study, although it could be written more clearly. I wonder what is the purpose of getting such a large panel, as getting consensus might not need so many people involved. The authors should consider data saturation, and the effort needed to recruit so many people.
- Lacking line numbers or page numbers to give specific comments.
- Not sure why the authors change between the terms youth and young people. The target audience is 13-17 year old's these are considered as adolescents (Ref 1). Change all terminology to reflect that to reduce the words and being consistency in the writing.

**Specific comments**

- First reference does not require 3 references. Choose one.
- 2nd sentence requires a reference to understand where the authors perspective come from.
- Last sentence of the first paragraph, is unsubstantiated by the previous references. It is more about how to improve on qol and strength. But nothing about other areas of health, i.e. social or mental.
- 2nd paragraph, is the study being carried out in the UK? Why not refer to the Irish PA guidelines? Most of Smith et Al study, includes children with CP, so although it makes sense in one way to use Smith, it is not completely relevant for the context where the WHO guidelines have been adopted.
- Reference from activity allowance needs more context and quality of evidence. Previous studies used quality essays to measure CP and PA and outcomes, activity allowance is less rigorous.
- 3rd paragraph seems disjointed. Try to revise the elements of the ICF. See recent review on the ICF CY .
- 4th, by the background, it is not evident that the are barriers. If this is the focus, authors need to write more about barriers. Many literature reviews have been missing.
- Sample size: Reference is needed for the way if calculating participation snapshot.
- Write ms in full the first time
- Recruitment and data connection sections are well written.
- How does your ethics consider the use of country data?
- It is not clear about the participation snapshot. Is it the cape that is being used? If so, explain for the survey is converted into an instrument for the Dephi study.
- Even after reading the Dephi study section, it is still not clear how cape is used.
- Suggest to check with your data management officer if withdrawals can still have their data used, even if it is deidentified
- Statistical models seem rather ambitious with 100 participants and six variables
- The f words should be written out on full. It is not clear how they would be used. It should be introduced in the introduction.

Is the study design appropriate for the research question?

Partly

Is the rationale for, and objectives of, the study clearly described?

Partly

Are sufficient details of the methods provided to allow replication by others?

Partly

Are the datasets clearly presented in a useable and accessible format?

Not applicable

Reviewer Expertise:

adapted physical activity, health promotion, adolescents and children with disabilities, health promotion, psychology of exercise, public health, statistics, epidemiology

I confirm that I have read this submission and believe that I have an appropriate level of expertise to confirm that it is of an acceptable scientific standard, however I have significant reservations, as outlined above.

## References

[1] : The age of adolescence. *Lancet Child Adolesc Health* .2018;2(3) : 10.1016/S2352-4642(18)30022-1 223-228 10.1016/S2352-4642(18)30022-1 30169257

# Response

**Published:** 2024-03-20

**Authors:** Ailish Malone

**Affiliations:** Royal College of Surgeons in Ireland, Ireland

We thank the reviewer for the constructive feedback on this protocol. We now address each point in responses below the review comment.

**Title is very long. Suggest to shorten. Remove the youth experience matters, or combine the next two parts together.**

**Response:** We agree the title is long and adjusted it slightly to shorten while still retaining the key information on the subject matter, protocol for two studies, and the project working title. The title is now “Participation in physical activity by adolescents with physical disability: Protocol for a national participation snapshot and Delphi consensus study (“Youth Experience Matters”)”

**General comments**

- This is an interesting study and highlight ways to encourage people with disabilities to be the voices in research. It is a much needed area in research and can provide useful insights to help provide tailored programs for people with disabilities. The methods seem suitable for the study, although it could be written more clearly. I wonder what is the purpose of getting such a large panel, as getting consensus might not need so many people involved. The authors should consider data saturation, and the effort needed to recruit so many people.
  - **Response:** While other consensus studies have used smaller panels and we acknowledge the work involved in recruiting 100 participants, we based this decision on a sample size calculation informed by data from multiple data sources (Census, National Physical and Sensory Disability Database) and considered it appropriate due to the anticipated heterogeneity in perspectives from adolescents with disability, who are a diverse group. The target sample size equates to approximately 5% of the total population of 13-17 year olds (estimated to be 2,000 adolescents) with physical disability in Ireland and is comparable with other Delphi studies cited in this section. We anticipate attrition between Delphi rounds, so the final consensus may not involve all 100 participants (though they will all get the opportunity to contribute). 
- Lacking line numbers or page numbers to give specific comments.
  - **Response:** We added line numbers to the Word version (unsure if these will track across to online version).
- Not sure why the authors change between the terms youth and young people. The target audience is 13-17 year old's these are considered as adolescents (Ref 1). Change all terminology to reflect that to reduce the words and being consistency in the writing.
  - **Response:** We changed all instances of “youth”, “young people” and other variations to “adolescents” for consistency.

**Specific comments**

- First reference does not require 3 references. Choose one.
  - **Response: **We selected the WHO reference to support this first statement.
- 2nd sentence requires a reference to understand where the authors perspective come from.
  - **Response: **This sentence intends to lead to the next points, which synthesise benefits of engagement in PA from a selection of indicative studies. For clarity, we have added “as demonstrated by the following evidence.”
- Last sentence of the first paragraph, is unsubstantiated by the previous references. It is more about how to improve on qol and strength. But nothing about other areas of health, i.e. social or mental.
  - **Response:** We cite the work of Maher et al (2016) showing higher levels of happiness in adolescents with CP who were physically active. We acknowledge that mental health is a complex construct but nonetheless argue that higher levels of happiness supports the point of benefit across multiple domains of health.
- 2nd paragraph, is the study being carried out in the UK? Why not refer to the Irish PA guidelines? Most of Smith et Al study, includes children with CP, so although it makes sense in one way to use Smith, it is not completely relevant for the context where the WHO guidelines have been adopted.
  - **Response: **This study is being undertaken in the Republic of Ireland. We added the Irish PA guidelines to the beginning of this paragraph, noting a recent proposed update. As our study focuses on adolescents with physical disability, we include the Smith guidelines as they are specific to this population.
- Reference from activity allowance needs more context and quality of evidence. Previous studies used quality essays to measure CP and PA and outcomes, activity allowance is less rigorous.
  - **Response:** We agree the Activity Alliance survey is not a peer-reviewed source of evidence and therefore less rigorous. We have added a point to indicate this is a public engagement, non-peer reviewed source.
- 3rd paragraph seems disjointed. Try to revise the elements of the ICF. See recent review on the ICF CY.
  - **Response:** To make this clearer, we added a statement to introduce the ICF component of participation as the focus of the study. We include a reference to the ICF for readers to put this in context. We explain, using findings from other studies, that the social connectedness of participation in physical activity may have a sustaining effect on that activity.
- 4th, by the background, it is not evident that the are barriers. If this is the focus, authors need to write more about barriers. Many literature reviews have been missing.
  - **Response: **We agree that the literature on physical activity in people with disability is far more extensive than the points in this background. The project aims to focus on priorities for enhancing physical activity participation, and as such, is not focused specifically on barriers, though barriers are relevant to the priorities that might emerge. To make this clearer, we removed the point about barriers.
- Sample size: Reference is needed for the way if calculating participation snapshot.
  - **Response:** We added a reference to the resource used for this calculation.
- Write ms in full the first time
  - **Response: **MS is now defined in full (multiple sclerosis).
- Recruitment and data connection sections are well written.
- How does your ethics consider the use of country data?
  - **Response:** This study is recruiting participants from Ireland (Republic) only. There will not be data from other countries.
- It is not clear about the participation snapshot. Is it the cape that is being used? If so, explain for the survey is converted into an instrument for the Delphi study.
  - **Response:** This protocol comprises two interlinked, but distinct, studies. The first study, the participation snapshot, aims to describe current participation in physical activity by adolescents with physical disability, using the CAPE as the primary outcome measure. The second study, the Delphi, aims to establish priorities for physical activity participation. The CAPE is solely used in the participation snapshot and is not used in or for the Delphi study. After analysis of both studies, the findings of the participation snapshot and Delphi will be integrated to show a) current participation and b) priorities for how this can be improved. We have added this explanation to the [section]
- Even after reading the Delphi study section, it is still not clear how cape is used.
  - **Response:** this comment is addressed above.
- Suggest to check with your data management officer if withdrawals can still have their data used, even if it is deidentified
  - **Response:** We checked this with our Data Protection Officers. Once data are de-identified, it is not possible to withdraw them from the dataset as there is no link with the identifiable person. However, as the study design enables participants to effectively “opt in” to each Delphi round by email invitation, they can simply choose not to complete subsequent rounds. For the purposes of data minimisation, we do not have tracking in place to know which email invitations progress to completed surveys.
- Statistical models seem rather ambitious with 100 participants and six variables
  - **Response:** We have amended our statistical plan. Rather than exploring for evidence of interaction between predictor and dependent variables, we will now describe the findings of CAPE grouped by relevant participant characteristics (gender, underlying medical diagnosis, mobility, hand function, geography of place of residence).
- The f words should be written out on full. It is not clear how they would be used. It should be introduced in the introduction.
  - **Response:** We added an explanation of the F-words conceptualisation of the ICF to paragraph 3 of the introduction. Consistent with the aim of our study, we focused on the F-word “friends” as the conceptualisation of participation. Later, in Methods / Statistical Analysis, we explain that the F-words conceptualisation will be used as a framework for analysis of the free text responses to open-ended questions in the Delphi study.

# Reviewer report: Reviewer response for version 1

**DOI:** 10.21956/hrbopenres.15031.r37375

**Published:** 2024-02-16

**Authors:** Caitriona Cunningham

**Affiliations:** 1 School of Public Health, Physiotherapy and Sports Science, University College Dublin, Dublin, Ireland

Thank you for the invitation to review this protocol paper. An important  study has been proposed and overall a comprehensive and detailed protocol  is presented with well thought out  methods described. The authors are to be  commended for embarking on this major study.  

 The paper opens with an overview of evidence regarding the need for children and adolescents, with physical disability, to be physically active and makes the case for conducting the proposed  study. Ethics approval has been obtained.  Overall, the methods are clearly described  with a snapshot survey of adolescents and a subsequent Delphi study proposed.  PPI  is described. Sample size is justified. Outcome measures are specified. Recruitment mechanisms are described  with retention and participant withdrawal issues also considered.  Data analysis and management  mechanisms are also described.

 Some clarifications are required as outlined below, most importantly clarification in relation to what priorities are being established and  with  documentation of more specific study objectives  recommended.

**Main Points for** **Revision/** **Clarification**

**Aim and Objectives**

 Understand that study will capture the  perspectives of young [people with disability on Physical Activity (PA)  participation but  this is a very broad aim. Adding  a series of  specific objectives would help clarify (eg. to explore/ identify barriers to PA participation, to classify barriers/ facilitators using framework- under individual/ environmental/other socioecological domains?)

**Priorities**

 Top 10 Priorities -  for  what ? to facilitate PA participation of young with disability? Need to state if this is  the goal. What are priorities of  young people with disability when it comes to PA?

 Priorities as to what needs to change/ happen  in Ireland/ in service provision/in attitudes? -  research/ policy/ action/ national priorities?   priorities to be actioned by whom?

 This is not clear in abstract/paper 

 Crosscheck for clarity wherever priorities are mentioned in paper.

 Refer to both 10 ‘choices’ and 10 ‘priorities’ at different points – confusing.

**Other Points for** **Revision/** **Clarification**

**Abstract**

*'disability diagnosis'* - Please reword. As authors illustrate elsewhere in paper, disability is not a diagnosis but  results from a complex interplay of factors which may  include an underlying  medical diagnosis -  this needs to be demonstrated better  (social model of disability)  in wording used  here.

 Also clarify  in other paper sections eg.  Participants and Eligibility section – do say ‘cause of disability’ but it’s never just the medical diagnosis - perhaps use ‘ underlying medical  diagnosis’ as term ?

*'what really matters for participating'* - suggest reword  as ambiguous currently - could mean what encourages participation or what are benefits of participation- now obviously these may  overlap?    Are authors referring to what encourages participation? Be more explicit.   (see intro too)

 Top 10 Priorities-  not clear in abstract

**Introduction**

*Participation, defined in the ICF ......is crucial to sustaining meaningful engagement in PA*. -  Recommend rewording as  participation  more or less =   'engagement',  so  sentence doesn't  fully make sense

*'Adolescence is a significant time of change in participation*' Be explicit  that Imms' paper/s refers  specifically to children with disability.

*Participation is not only a desirable goal from the perspective of self-efficacy but also that enabling participation in PA, self-determined by individual choice, is a potentially impactful public health intervention to sustain PA for better future health.  (* *Imms et al., 2017* *). un*wieldy sentence that doesn't fully make sense  - may reflect source paper wording  but recommend reword/ replace 

 I *t is evident that young people with physical disability experience multiple barriers to PA participation and have difficulties in attaining sufficient PA to meet guideline recommendations.*

 Is it possible to provide a reference to support that statement or  at least make a more explicit  link  with   previous introduction content ?

**Aim**

 Seem to be stating aim in different places in different ways - crosscheck  across abstract and paper sections – again recommend add objectives.

 Recommend that remove last line of intro - *T* *his study aims to understand perspectives on PA of young people with a physical disability.*

 -as  author  goes on to state Aim in next section and  thus duplication.

**Priorities**

 As highlighted  previously, priorities require clarification.

**Methods**

**PPI**

 How many members are there on the  PPI panel?  - adult/ child numbers ?

 Parents – add **and/or guardians?**

**Literacy considerations for survey**

 In addition to commendable PPI, will any specific child  literacy guidelines be followed in designing the survey?

**Sample**

**'National'?**

  Consider all readers and especially international readership – add a little more detail as reassurance that sample will be  nationally representative?  Could it be biased towards location of lead clinical centre? Does this centre serve  young people  from all over country?  Public/ Private services ?  CDNTS -  are  these and other clinical services geographically spread ? Urban/Rural? Socio-demographic areas?  Will a  range of levels of disability be represented?

 Is it  necessary to use additional  ‘WP 1’ and ‘WP 2’ headers in methods? - maybe clearer to be consistent and just use ‘Participant Snapshot Survey’ and  ‘Delphi Study’ throughout ?

 Under  what’s currently headed as WP1-Participation Snapshot, recommend adding ‘online, paper and/ or interview led  survey’  

 as part of Study Design description versus just saying CS study or at least indicate that it’s a ‘survey’ +/- interviews  that is being proposed here.

**Sample Size**

*Taking 2,000 eligible participants as the target population, recruiting 92 participants will give a confidence interval of ±10% for calculation of the Participation Snapshot.* – revisit this sentence –  add more detail  - so estimating that 5%  (n=100) of target population will be recruited

**CAPE**

 Worth describing if it has a specific ‘Physical activity domain’ …..  any reference  to support its use in capturing youth  PA levels ? A reference is provided regarding  it  being used  previously but clarify its use specifically for capturing childhood PA.

*Extrapolating from Census 2016, 411,519 people living in Ireland will be age 13–17 in 2022 (* *CSO, 2016* *)... *maybe reword so better integrated  into paper as we're now in 2024- change tense - *that x **would be** aged between 13 and 17…*

 Dates November 2022 and March 2024.  Does start date need revisiting ? Protocol paper ideally  should be written/published  prior to commencement of data collection.

**Analysis of Delphi  Study Data**

 Good description of thematic analysis of written data

 What about describing analysis of likert scale data ? (ie. quantitative data)

 Would query use of ‘ *regression analysis’*  to explore for differences in  profile of participants  at different rounds.  Recommend that  proposed analysis is revised here.

**Figure 3** – Don’t believe this is  a necessary graphic but  authors can decide.

 I look forward to hearing more about this study and its results.

Is the study design appropriate for the research question?

Yes

Is the rationale for, and objectives of, the study clearly described?

Partly

Are sufficient details of the methods provided to allow replication by others?

Yes

Are the datasets clearly presented in a useable and accessible format?

Not applicable

Reviewer Expertise:

musculoskeletal health, promotion of  physical activity and exercise for prevention and management of disease, disability inclusion

I confirm that I have read this submission and believe that I have an appropriate level of expertise to confirm that it is of an acceptable scientific standard, however I have significant reservations, as outlined above.

## References

[1] : Participation, both a means and an end: a conceptual analysis of processes and outcomes in childhood disability. *Dev Med Child Neurol* .2017;59(1) : 10.1111/dmcn.13237 16-25 10.1111/dmcn.13237 27640996

# Response

**Published:** 2024-03-20

**Authors:** Ailish Malone

**Affiliations:** Royal College of Surgeons in Ireland, Ireland

We thank the reviewer for the constructive comments. We now address each point in responses below the review comments.

 Thank you for the invitation to review this protocol paper. An important  study has been proposed and overall a comprehensive and detailed protocol  is presented with well thought out  methods described. The authors are to be  commended for embarking on this major study.  

- **Response:** Thank you for reviewing this protocol. We appreciate the feedback on the importance of the study, suitable methods and comprehensive protocol.

**Main Points for** **Revision/Clarification**

**Aim and Objectives**

 Understand that study will capture the perspectives of young [people with disability on Physical Activity (PA) participation but this is a very broad aim. Adding a series of specific objectives would help clarify (eg. to explore/ identify barriers to PA participation, to classify barriers/ facilitators using framework- under individual/ environmental/other socioecological domains?)

- **Response: **We rephrased the aim to “To establish consensus among adolescents with physical disability regarding their priorities for **enhancing** their physical activity participation”. We added specific objectives.

**Priorities**

 Top 10 Priorities -  for  what ? to facilitate PA participation of young with disability? Need to state if this is  the goal. What are priorities of  young people with disability when it comes to PA?

 Priorities as to what needs to change/ happen  in Ireland/ in service provision/in attitudes? -  research/ policy/ action/ national priorities?   priorities to be actioned by whom?

 This is not clear in abstract/paper 

 Crosscheck for clarity wherever priorities are mentioned in paper.

 Refer to both 10 ‘choices’ and 10 ‘priorities’ at different points – confusing.

- **Response:** We agree the mentions of priorities could be clearer. We rephrased to “priorities for **enhancing** their physical activity participation”. Under implications, we describe that these priorities could then be used to inform future intervention development, policy and practice. Under subheading “Dissemination and knowledge translation”, we describe how we will identify ways to share the findings for impact on policy and practice. Some of the reviewer’s suggested targets may well become part of the work that follows from the priorities.  We added a statement to that effect.

**Other Points for** **Revision/Clarification**

**Abstract**

*'disability diagnosis'* - Please reword. As authors illustrate elsewhere in paper, disability is not a diagnosis but  results from a complex interplay of factors which may  include an underlying  medical diagnosis -  this needs to be demonstrated better  (social model of disability)  in wording used  here.

 Also clarify  in other paper sections eg.  Participants and Eligibility section – do say ‘cause of disability’ but it’s never just the medical diagnosis - perhaps use ‘ underlying medical  diagnosis’ as term ?

- **Response: **We agree this language could be more consistently aligned to the social model of disability and ICF. We rephrased to “underlying medical diagnosis”

*'what really matters for participating'* - suggest reword  as ambiguous currently - could mean what encourages participation or what are benefits of participation- now obviously these may  overlap?    Are authors referring to what encourages participation? Be more explicit.   (see intro too)

- **Response: **We agree this was ambiguous. Following from the previous review points, we now consistently state the aim to establish consensus on priorities for enhancing physical activity participation. 

Top 10 Priorities-  not clear in abstract

- **Response:** Consistent with the manuscript, this now states “top 10 priorities for enhancing physical activity participation”.

**Introduction**

*Participation, defined in the ICF ......is crucial to sustaining meaningful engagement in PA*. -  Recommend rewording as  participation  more or less =   'engagement',  so  sentence doesn't  fully make sense

- **Response: **We have rephrased this more simply to “participation could be crucial to sustaining PA”

*'Adolescence is a significant time of change in participation*' Be explicit  that Imms' paper/s refers  specifically to children with disability.

- **Response:** We add this qualifier as follows: “Adolescence is a significant time of change in participation, *when adolescents with physical disability* shift away from recreational and physical activities towards more social participation (Imms & Adair, 2017).”

*Participation is not only a desirable goal from the perspective of self-efficacy but also that enabling participation in PA, self-determined by individual choice, is a potentially impactful public health intervention to sustain PA for better future health.  (Imms et al., 2017). un*wieldy sentence that doesn't fully make sense  - may reflect source paper wording  but recommend reword/ replace 

- **Response: **We removed this sentence.

I *t is evident that young people with physical disability experience multiple barriers to PA participation and have difficulties in attaining sufficient PA to meet guideline recommendations.*

 Is it possible to provide a reference to support that statement or  at least make a more explicit  link  with   previous introduction content ?

- **Response: **We revised this statement to create a clearer link with the previous introduction and lead more clearly into the study aims. It now reads “To determine how a participation-based approach could improve physical activity, it is necessary to measure current participation and understand the perspectives of adolescents themselves on the factors that could enhance participation.”

**Aim**

 Seem to be stating aim in different places in different ways - crosscheck  across abstract and paper sections – again recommend add objectives.

- **Response:** We revised the aims and added objectives. We crosschecked the versions of the aims and they are now congruent across all sections of the paper.

Recommend that remove last line of intro - *This study aims to understand perspectives on PA of young people with a physical disability.*

 -as  author  goes on to state Aim in next section and  thus duplication.

- **Response:** We agree this line was unnecessary and removed it.

**Priorities**

 As highlighted  previously, priorities require clarification.

- **Response:** We explain that these priorities are “factors that could enhance their participation in PA”.

**Methods**

**PPI**

 How many members are there on the  PPI panel?  - adult/ child numbers ?

- **Response:** There were three adolescents and three parents / guardians on the PPI panel. This is now stated in the manuscript.

Parents – add **and/or guardians?**

- **Response: **We added parents / guardians wherever parents is stated.

**Literacy considerations for survey**

 In addition to commendable PPI, will any specific child  literacy guidelines be followed in designing the survey?

- **Response: **We did not follow specific literacy guidelines however we did follow local practice in the clinical site, which provides children’s services. In addition to PPI input we also piloted the survey with 6 children age 10-17, who were not eligible for the study, and sought their feedback on readability.

**Sample**

**'National'?**

  Consider all readers and especially international readership – add a little more detail as reassurance that sample will be  nationally representative?  Could it be biased towards location of lead clinical centre? Does this centre serve  young people  from all over country?  Public/ Private services ?  CDNTS -  are  these and other clinical services geographically spread ? Urban/Rural? Socio-demographic areas?  Will a  range of levels of disability be represented?

- **Response:** We considered the geographical distribution in identifying the study gatekeepers and sharing information. The lead clinical site serves young people from all over Ireland in its specialist services, and additionally has smaller clinics in the south east and west of Ireland. The other clinical partner provides children’s services in 11 of 26 counties, and in all four provinces. Eligibility criteria include all levels of disability and this was represented in the images used in our recruitment material and PILs, which included wheelchair users and ambulant young people.

Is it  necessary to use additional  ‘WP 1’ and ‘WP 2’ headers in methods? - maybe clearer to be consistent and just use ‘Participant Snapshot Survey’ and  ‘Delphi Study’ throughout ?

 Under  what’s currently headed as WP1-Participation Snapshot, recommend adding ‘online, paper and/ or interview led  survey’  

 as part of Study Design description versus just saying CS study or at least indicate that it’s a ‘survey’ +/- interviews  that is being proposed here.

- **Response:** We removed the “WP” prefix and refer to Participation Snapshot and Delphi throughout. The Participation Snapshot is now described as “a cross-sectional study, comprising an online, paper and/ or interview led survey”

**Sample Size**

*Taking 2,000 eligible participants as the target population, recruiting 92 participants will give a confidence interval of ±10% for calculation of the Participation Snapshot.* – revisit this sentence –  add more detail  - so estimating that 5%  (n=100) of target population will be recruited

- **Response:** We added this statement “100 participants… *estimated to be 5% of the target population*” to the sample size description.

**CAPE**

 Worth describing if it has a specific ‘Physical activity domain’ …..  any reference  to support its use in capturing youth  PA levels ? A reference is provided regarding  it  being used  previously but clarify its use specifically for capturing childhood PA.

- **Response: **We followed the methods of Woodmansee et al. (2016) who used this 16-item subsection of the CAPE in measuring participation in physical activity in 163 children age 6-17 years with physical, intellectual or sensory disability. We added this detail to the manuscript.

*Extrapolating from Census 2016, 411,519 people living in Ireland will be age 13–17 in 2022 (CSO, 2016)... *maybe reword so better integrated  into paper as we're now in 2024- change tense - *that x **would be** aged between 13 and 17…*

- **Response: **We have changed the tense for clarity.

Dates November 2022 and March 2024.  Does start date need revisiting ? Protocol paper ideally  should be written/published  prior to commencement of data collection.

- **Response:** The protocol was written for grant submission in October 2021 and finalised in December 2022. Following approval by Ethics, recruitment commenced in March 2023. We have updated the start date. This protocol paper was submitted to HRBOpen in May 2023, while recruitment was ongoing.

**Analysis of Delphi  Study Data**

 Good description of thematic analysis of written data

 What about describing analysis of likert scale data ? (ie. quantitative data)

- **Response:** We added a description of analysis of Likert scale data

Would query use of ‘ *regression analysis’*  to explore for differences in  profile of participants  at different rounds.  Recommend that  proposed analysis is revised here.

- **Response:** We changed this to a descriptive analysis of differences in important participant characteristics that might affect interpretation of findings between rounds.

**Figure 3** – Don’t believe this is  a necessary graphic but  authors can decide.

- **Response:** We agree Figure 3 does not add to the understanding of the Methods however we have retained it as an example of engagement with adolescents for a research study, which may be of interest to colleagues planning other studies.
